# High-level Relatedness among *Mycobacterium abscessus* subsp. *massiliense* Strains from Widely Separated Outbreaks

**DOI:** 10.3201/eid2003.131106

**Published:** 2014-03

**Authors:** Hervé Tettelin, Rebecca M. Davidson, Sonia Agrawal, Moira L. Aitken, Shamira Shallom, Nabeeh A. Hasan, Michael Strong, Vinicius Calado Nogueira de Moura, Mary Ann De Groote, Rafael S. Duarte, Erin Hine, Sushma Parankush, Qi Su, Sean C. Daugherty, Claire M. Fraser, Barbara A. Brown-Elliott, Richard J. Wallace, Steven M. Holland, Elizabeth P. Sampaio, Kenneth N. Olivier, Mary Jackson, Adrian M. Zelazny

**Affiliations:** University of Maryland School of Medicine, Baltimore, Maryland, USA (H. Tettelin, S. Agrawal, E. Hine, S. Parankush, Q. Su, S.C. Daugherty, C.M. Fraser);; National Jewish Health, Denver, Colorado, USA (R.M. Davidson, N.A. Hasan, M. Strong);; University of Washington, Seattle, Washington, USA (M.L. Aitken);; National Institutes of Health, Bethesda, Maryland, USA (S. Shallom, S.M. Holland, E.P. Sampaio, K.N. Olivier, A.M. Zelazny);; University of Colorado Denver, Aurora, Colorado, USA (N.A. Hasan, M. Strong);; Colorado State University, Fort Collins, Colorado, USA (V. Calado Nogueira de Moura, M.A. De Groote, M. Jackson);; Universidade Federal do Rio de Janeiro, Rio de Janeiro, Brazil (R.S. Duarte);; University of Texas Health Northeast, Tyler, Texas, USA (B.A. Brown-Elliott, R.J. Wallace Jr.)

**Keywords:** *Mycobacterium abscessus*, *Mycobacterium abscessus* subsp. *massiliense*, bacteria, tuberculosis and other mycobacteria, strains, cystic fibrosis, relatedness, outbreaks, geographically distant outbreaks, United States, United Kingdom

## Abstract

Three recently sequenced strains isolated from patients during an outbreak of *Mycobacterium abscessus* subsp. *massiliense* infections at a cystic fibrosis center in the United States were compared with 6 strains from an outbreak at a cystic fibrosis center in the United Kingdom and worldwide strains. Strains from the 2 cystic fibrosis outbreaks showed high-level relatedness with each other and major-level relatedness with strains that caused soft tissue infections during an epidemic in Brazil. We identified unique single-nucleotide polymorphisms in cystic fibrosis and soft tissue outbreak strains, separate single-nucleotide polymorphisms only in cystic fibrosis outbreak strains, and unique genomic traits for each subset of isolates. Our findings highlight the necessity of identifying *M. abscessus* to the subspecies level and screening all cystic fibrosis isolates for relatedness to these outbreak strains. We propose 2 diagnostic strategies that use partial sequencing of *rpoB* and *secA1* genes and a multilocus sequence typing protocol.

Nontuberculous mycobacteria (NTM) and, in particular, the *Mycobacterium abscessus* group are recognized as emerging respiratory pathogens among patients with cystic fibrosis. Reports from the United States, France, and Israel have shown that the *M. abscessus* group accounts for a major proportion of NTM infections in patients with cystic fibrosis; prevalence rates range from 16% to 48% (*1*–*3*).

Previous studies have indicated great diversity within *M. abscessus* group strains among cystic fibrosis patients, suggesting independent acquisitions of NTM from the environment (*2*,*4*). However, suspicion of patient-to-patient transmission arose with the recent report of an outbreak of respiratory infection with *M. abscessus* subsp. *massiliense* at a cystic fibrosis center in Seattle, Washington, USA (*5*). The index case-patient and 4 additional patients all had multidrug-resistant isolates with resistance to amikacin and clarithromycin. All 5 strains were indistinguishable by repetitive unit sequence–based PCR patterns and pulsed-field gel electrophoresis analysis, which led to initiation of whole-genome sequencing. In a separate, recent study, whole-genome sequencing and epidemiologic analysis provided strong support for patient-to-patient transmission in 2 clustered outbreaks of *M. abscessus* subsp. *massiliense* at the Papworth Hospital Cystic Fibrosis Centre (Cambridge, UK) (*6*). Isolates from both clusters showed resistance to clarithromycin, and isolates from one of the clusters also had mutations conferring resistance to amikacin.

The availability of whole-genome sequences from different *M. abscessus* subsp. *massiliense* outbreaks, as well as unrelated strains, provides an unprecedented opportunity for multigenome comparisons. We conducted a genomic study of 3 recently sequenced strains from the Seattle cystic fibrosis outbreak, including the index strain, and compared them with representative strains from the Papworth cystic fibrosis outbreak, as well as with available strains from the United Kingdom, the United States, Brazil, South Korea, France, and Malaysia (Table 1**).** We found high-level relatedness among strains from the 2 geographically distant outbreaks in Seattle and Papworth. We also identified shared and unique genomic traits for strains from both cystic fibrosis outbreaks and for those from an outbreak of soft tissue infections in Brazil.

**Table 1:** Twenty-four *Mycobacterium abscessus* group strain genomes analyzed for genetic relatedness*

Subspecies/strain	Country	Outbreak	GenBank accession no.	Reference
Mm/2u	UK	Papworth	NA	(*6*)
Mm/12c	UK	Papworth	NA	(*6*)
Mm/14h	UK	Papworth	NA	(*6*)
Mm/19f	UK	Papworth	NA	(*6*)
Mm/20h	UK	Papworth	NA	(*6*)
Mm/28c	UK	Papworth	NA	(*6*)
Mm/2B-0107	USA	Seattle	AKUN00000000	This study
Mm/MAB_082312_2258	USA	Seattle	AYTA00000000	This study
Mm/MAB_091912_2446	USA	Seattle	AYTF00000000	This study
Mm/CRM-0020	Brazil	Rio de Janeiro	ATFQ00000000	(*7*)
Mm/GO-06	Brazil	Goiás	CP003699	(*8*)
Mm/47J26	UK	Not applicable	AGQU01000000	(*9*)
Mm/M18	Malaysia	Not applicable	AJSC01000000	(*10*)
Mm/M115	Malaysia	Not applicable	AJLZ00000000	(*11*)
Mm/M139	Malaysia	Not applicable	AKVR01000000	(*12*)
Mm/M154	Malaysia	Not applicable	AJMA01000000	(*13*)
Mm/Asan 50594	South Korea	Not applicable	CP004374–CP004376	(*14*)
Mm/1S-151–930	USA	Not applicable	AKUI00000000	This study
Mm/5S-0817	USA	Not applicable	AKUB00000000	This study
Mm/CCUG 48898^T^	France	Not applicable	AKVF01000000	(*15**,**16*)
Ma/CF	France	Not applicable	CAHZ00000000	(*17*)
Ma/ATCC 19977^T^	USA	Not applicable	CU458896,CU458745	(*18*)
Mb/BD^T^	France	Not applicable	AHAS00000000	(*19*)
Mb/M24	Malaysia	Not applicable	AJLY00000000	(*20*)

*Mm, *M. abscessus* subsp. *massiliense*; NA, not available; Ma, *M. abscessus* subsp. *abscessus*; Mb, *M. abscessus* subsp. *bolletii*.

## Materials and Methods

### Sequence Analysis of Outbreak Strains

A subset of 6 isolates (2u, 12c, 14h, 19f, 20h, and 28c) representing the breadth of genomic diversity observed within the Papworth cystic fibrosis outbreak clusters 1 and 2 (*6*) were selected. Illumina sequencing reads from each of these isolates were assembled into sets of contigs by using Velvet software (*21*). These contigs were combined with draft genome sequences of the Seattle cystic fibrosis outbreak and available whole-genome sequences of *M. abscessus* subsp. *massiliense* (Table 1) and subjected to whole-genome multiple sequence alignments by using Mugsy software (*22*). Core segments of the alignment that are shared among all isolates included in the analysis were identified and concatenated by using Phylomark software (*23*). Concatenated nucleotide sequences, including single-nucleotide polymorphisms (SNPs), were then used for construction of a neighbor-joining phylogenetic tree by using MEGA software (*24*). The use of microbial samples and data was approved by the ethics committees at each of the institutions involved.

To replicate data from the Papworth cystic fibrosis outbreak clusters 1 and 2 (*6*) by using a similar approach, we mapped sequencing reads from the subset of 6 Papworth isolates, together with reads with from the 3 Seattle cystic fibrosis isolates and soft tissue strain CRM-0020 from Brazil (Table 1), onto the *M. abscessus* type strain ATCC 19977^T^ reference genome by using BWA software (*25*). Variants, including SNPs, were called by using GATK software (*26*) and filtered for quality. The SNP panel was used for construction of a neighbor-joining phylogenetic tree by using MEGA software. The resulting tree replicated the topology of clusters 1 and 2 and showed that the Seattle isolates are most closely related to cluster 2.

### PCR and In Silico PCR

Standard PCR and sequencing strategies were used to amplify and analyze partial sequences of the *rpoB* (723 bp) (*27**,**28*) and *secA1* (465 bp) (*29*) genes. In addition, a multilocus sequence typing (MLST) scheme (*29**,**30*), including primers to 13 housekeeping genes (*cya*, *gdhA*, *argH*, *glpK*, *gnd*, *murC*, *pgm*, *pknA*, *pta*, *pur*, *rpoB*, *hsp65*, and *secA1*) was used to conduct electronic PCR on the panel of 20 *M. abscessus* subsp. *massiliense* genomes (Table 1). Published forward primers for *cya* and *gdhA* (*30*) did not amplify in silico for some *M. abscessus* subsp. *massiliense* strains; therefore, the following new primers conserved across the *M. abscessus* group were used: cya_F_new 5′-GCC TGC GTA AGG GTG ATG-3′ and gdhA_F_new 5′-GTG AAG CTC GCC GCC TGC-3′. Alleles from each gene were extracted and concatenated for each genome, the panel of concatenated sequences was aligned by using ClustalW software (*31*), and the core segments of the alignment were used for construction of a neighbor-joining phylogenetic tree by using MEGA software.

## Results

### Phylogenetic Characteristics of Outbreak Strains

**Figure 1:**
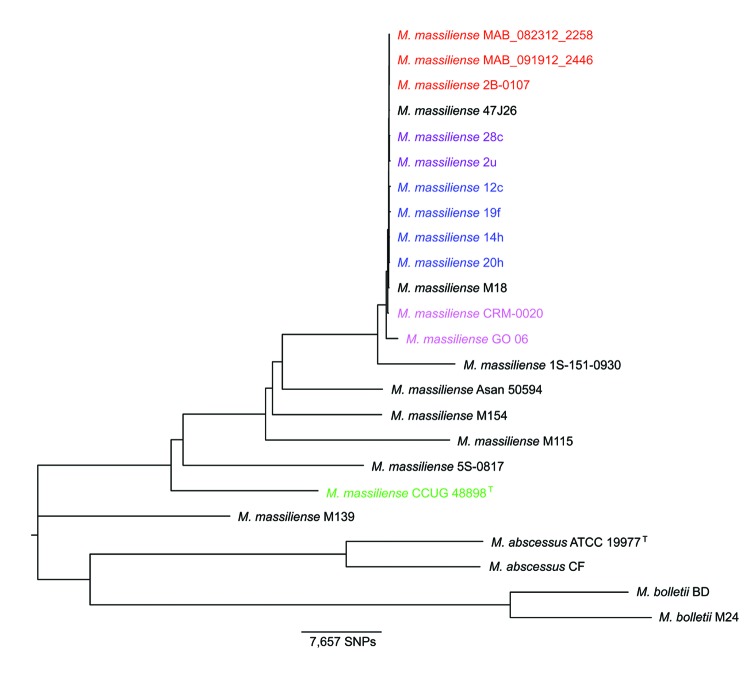
Neighbor-joining phylogenetic tree based on whole-genome multiple alignment of 24 *Mycobacterium abscessus* group genomes. Genomes in Table 1 were aligned by using Mugsy (*22*), core segments of the alignment were identified by using Phylomark (*23*), and resulting concatenated nucleotide sequences were used for construction of the midpoint-rooted neighbor-joining phylogenetic tree by using MEGA (*24*). Strains from an outbreak of *M. abscessus* subsp. *massiliense* infections at a cystic fibrosis center in Seattle, Washington, USA, are indicated in red; strains from an outbreak of *M. abscessus* subsp. *massiliense* infections at a cystic fibrosis center in Papworth, UK, are indicated in blue (cluster 1) and purple (cluster 2); strains from Brazil are indicated in magenta; and the *M. abscessus* subsp. *massiliense* type strain is indicated in green. Boostrap values obtained after 100 iterations were ≥97 for all nodes of the tree except 70 for the node separating strain M115 from the outbreak cluster and 40 and 41 for 2 nodes within the Papworth cluster 1 (*6*). SNPs, single-nucleotide polymorphisms.

A core genome phylogenetic tree (Figure 1) showed a tight cluster of the 3 Seattle cystic fibrosis outbreak strains. The Seattle cystic fibrosis cluster was closely related to the 2 cystic fibrosis clusters described for the Papworth outbreak (*6*) and the Birmingham, UK, cystic fibrosis isolate 47J26 (*9*). Furthermore, the Seattle and Papworth cystic fibrosis outbreak strains showed some relatedness to strains CRM-0020 and GO-06 derived strains (known collectively as BRA-100) isolated during an epidemic of soft tissue infections in Brazil (*32*) and the *M. abscessus* subsp. *massiliense* M18 strain from Malaysia (*10*).

The cumulative size of core segments of Mugsy alignments provides information on relatedness among groups of strains compared. The core genome reduces in size as more genomes are added; an expected major decrease occurs after addition of more distant strains to the group. The average genome size of cystic fibrosis outbreak strains was 4.81 Mb for Seattle (n = 3) and 4.97 Mb for Papworth (n = 6). The Seattle and Papworth cystic fibrosis outbreak strains (n = 9) shared a core genome of 4,264,844 nt, which is almost unchanged by including the Birmingham cystic fibrosis strain 47J26 (n = 10; 4,264,127 nt). Addition of the soft tissue outbreak strain CRM-0020 from Brazil (n = 11) (*32*) decreased the core to 4,231,390 nt, and adding the related outbreak strain GO 06 from Brazil (n = 12) (*8**,**33*), led to an additional decrease in the core genome to 4,043,718 nt. As expected, including unrelated available clinical *M. abscessus* subsp. *massiliense* strains (n = 20, including M139 with ambiguous subspecies taxonomic assignment (*12*), (Table 1), reduced the core genome size to 3,869,950 nt. Further addition of *M. abscessus* subsp. *abscessus* (n = 2) and *M. abscessus* subsp. *bolletii* (n = 2) genomes (Table 1) reduced the core to 3,828,656 nt.

### Genomes of Strains from Cystic Fibrosis and Soft Tissue Infection Outbreaks

The core genome of 10 strains representing the Papworth cystic fibrosis (n = 6), the Seattle cystic fibrosis (n = 3), and soft tissue CRM-0020 (n = 1) outbreaks comprised 4,231,390 nt. Strain GO 06 was excluded from the analysis because its genome harbors a large number of ambiguous nucleotides and an unusual hybrid appearance with fragments of *M. abscessus* subsp. *massiliense* and *M. abscessus* subsp. *abscessus* sequences. Strains 47J26 and M18, isolated from the sputum of a cystic fibrosis patient in Birmingham, UK, and a lymph node sample from a patient in Malaysia, respectively, were related to the outbreak strains (Figure 1). However, no information was available about any epidemiologic link between cystic fibrosis strain 47J26 to reported or unpublished outbreaks, and no clinical information was available about the patient from whom strain M18 was isolated. Therefore, both strains were excluded from the SNP analysis. Nevertheless, SNPs for these 3 strains at positions relevant to the outbreak strains are shown in the Technical Appendix.

**Figure 2:**
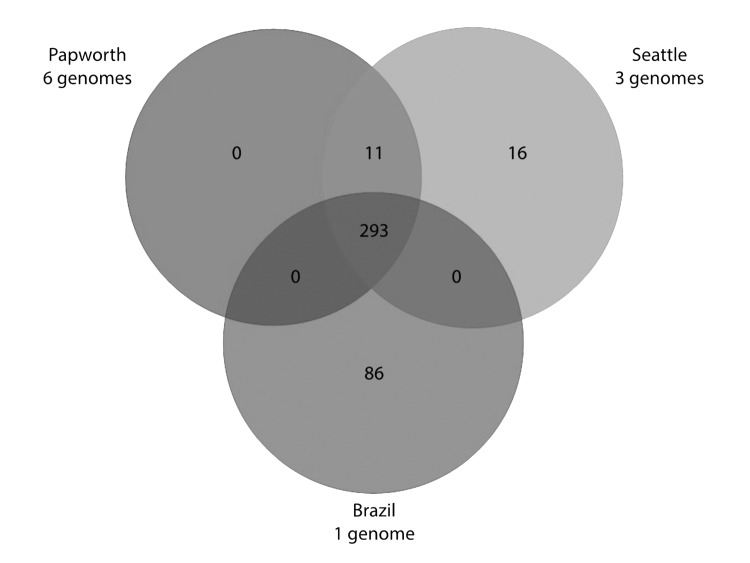
Venn diagram of core single-nucleotide polymorphisms (SNPs) shared by outbreak localities. Core segments of the Mugsy (*22*) alignment of the 20 *Mycobacterium abscessus* subsp. *massiliense* genomes (Table 1) were parsed for SNPs shared by different subsets of outbreak localities. Each field in the Venn diagram represents nucleotides that are identical among isolates of that field but different in other isolates represented on the diagram and non–outbreak-related *M. abscessus* subsp. *massiliense* strains 1S-151–0930, 5S-0817, M115, M139, M154, and the type strain CCUG 48898^T^. Details on SNPs and genes they affect are shown in the Technical Appendix.

A total of 293 identical SNPs in the core segments of Mugsy alignments were shared by the 10 outbreak strains but were different in available *M. abscessus* subsp. *massiliense* strains not related to outbreaks (Figure 2; Technical Appendix). Of the 293 SNPs, 95 gave rise to nonsynonymous mutations in several genes, including virulence factors (mammalian cell entry and yrbE proteins), transcriptional regulators (TetR family), and lipid metabolism genes (Technical Appendix). Eleven SNPs were shared only by Papworth and Seattle cystic fibrosis outbreak strains (n = 9), including nonsynonymous mutations in the preprotein translocase *secA1* and a putative lyase (Figure 2; Technical Appendix). Sixteen SNPs were shared only by the 3 Seattle cystic fibrosis outbreak strains, including nonsynonymous mutations in a mycobacterial large membrane protein (MmpL) family involved in lipid transport and virulence (*34*) and genes involved in amino acid and energy metabolism (Figure 2; Technical Appendix). Eighty-six SNPs were present only in strain CRM-0020 (soft tissue outbreak) from Brazil, including nonsynonymous mutations in an MmpL family protein; transcriptional regulators; and lipid, amino acid, and energy metabolism genes (Figure 2; Technical Appendix).

Having shown high-level relatedness among Papworth and Seattle cystic fibrosis outbreak strains and their relatedness to the soft tissue outbreak strains from Brazil, we also searched for genomic regions ≥200 nt outside the core genome that were specific to subsets of isolates. A single region of ≈11.5 kb was unique to the Papworth cystic fibrosis isolates (n = 6) and encompassed 2 conserved hypothetical proteins, 2 phage integrase family proteins, and an MmpL family protein. Alignment of the MmpL family protein with distinct MmpL proteins described above for the Seattle cystic fibrosis outbreak and the Brazil soft tissue outbreak showed diversity at several amino acid residues in all 3 proteins.

No region was unique to the Seattle cystic fibrosis isolates (n = 3). The soft tissue isolate CRM-0020 from Brazil harbored several large unique regions, including a previously described broad–host-range IncP-1β plasmid (*35*) and 3 regions (contigs) of 5 kb, 10.6 kb, and 79 kb of unknown origin encoding almost exclusively hypothetical proteins.

We also searched for polymorphisms associated with macrolide and aminoglycoside resistance. The Papworth cystic fibrosis and Seattle cytic fibrosis outbreak set of strains showed an A2058C/G mutation in 23S rRNA, which conferred macrolide resistance (*36*) (A2058G in strains 2u and 28c representative of Papworth cluster 2 and the Seattle strains). Strains 19f, 14h, 12c, and 28a, representative of Papworth cluster 1, and Seattle strains shared the A1408G mutations in 16S rRNA, which conferred aminoglycoside resistance (*37*). None of these mutations were found in the soft tissue outbreak strains CRM-0020 and GO 06 from Brazil or the M18 strain.

### Diagnostic Tools for Identification of Outbreak Strains

In light of the possibility of a common ancestor and/or intercontinental transmission of strains, we identified SNPs in genes commonly used for identification of mycobacteria and an MLST scheme that could be used by clinical laboratories to assess relatedness of newly isolated strains to this global cluster. In the first approach, we retrieved *rpoB* sequences from the 6 genomes of representative strains of the Papworth cystic fibrosis outbreak and performed partial sequencing of the *rpoB* gene for selected isolates from the Seattle cystic fibrosis outbreak. We then compared these sequences with those of isolates from the outbreak in Brazil and unrelated clinical isolates comprising *M. abscessus* subsp. *abscessus*, *massiliense*, and *bolletii*, as well as other rapidly growing mycobacteria.

By using the *rpoB* gene MAB_3869c from the *M. abscessus* subsp. *abscessus* type strain as a reference (Table 2) described in the BRA-00 outbreak isolates from Brazil (*32**,**33*), we showed that Seattle (n = 4) and Papworth (n = 6) cystic fibrosis isolates carried the 2 *rpoB* SNPs (C→T at position 2569 and T→C at position 2760. However, none of the *M. abscessus* subsp. *abscessus* or subsp. *bolletii* or other rapidly growing mycobacterial isolates outside the *M. abscessus* group harbored this 2-SNP *rpoB* signature (Table 2). The second SNP (T→C substitution at position 2760) was present in several strains, but the combination of both *rpoB* SNPs (C→T at position 2569 and T→C at position 2760) was not present. Most of the 26 *M. abscessus* subsp. *massiliense* strains not related to outbreaks tested did not harbor this 2-SNP *rpoB* signature. However, 4 strains harbored this signature (Table 2) (*29**,**38*).

**Table 2:** Detection of *rpoB* and *secA1* SNP signature in the *Mycobacterium abscessus* group and rapidly growing mycobacteria*

Strains	No. strains with SNP/no. tested (%)			
	*rpoB* T 2569	*rpoB* C 2760	*rpoB* T 2569 and *rpoB* C 2760	*secA1* T 820
MAB (CSU)	0/44 (0)	44/44 (100)	0/44 (0)	NT
MAB (NIH)	0/29 (0)	29/29 (100)	0/29 (0)	0/29 (0)
MMA non-outbreak strain (CSU)	1/14 (7)	1/14 (7)	0/14 (0)	NT
MMA non- outbreak strain (NIH)	4/12 (33)	10/ 12 (83)	4/12 (33)	2/12 (17)
MMA Seattle	4/ 4 (100)	4/4 (100)	4/4 (100)	3/3 (100)
MMA Brazil	9/ 9 (100)	9/9 (100)	9/9 (100)	NT
MBO (CSU)	0/11 (0)	11/11 (100)	0/11 (0)	NT
MBO (NIH)	0/2 (0)	2/2 (100)	0/2 (0)	0/2 (0)
Other RGM (CSU)	0/42 (0)	0/42 (0)	0/42 (0)	NT
MMA type strain	0/1 (0)	0/1 (0)	0/1 (0)	0/1 (0)
MMA In silico data†				
MMA UK	6/6 (100)	6/6 (100)	6/6 (100)	6/6 (100)
MMA Seattle	3/3 (100)	3/3 (100)	3/3 (100)	3/3 (100)
MMA Brazil	2/2 (100)	2/2 (100)	2/2 (100)	0/2 (0)
47J26	1/1 (100)	1/ 1 (100)	1/1 (100)	1/1 (100)
M18	1/1 (100)	1/1 (100)	1/1 (100)	1/1 (100)
1S-151–0930	1/1 (100)	1/1 (100)	1/1 (100)	0/1 (0)
5S-0817	0/1 (0)	1/1 (100)	0/1 (0)	0/1 (0)
M115	0/1 (0)	0/1 (0)	0/1 (0)	0/1 (0)
M139	0/1 (0)	1/1 (100)	0/1 (0)	0/1 (0)
M148	0/1 (0)	0/1 (0)	0/1 (0)	0/1 (0)
M154	0/1 (0)	0/1 (0)	0/1 (0)	0/1 (0)
M156	0/1 (0)	0/1 (0)	0/1 (0)	0/1 (0)
M159	0/1 (0)	0/1 (0)	0/1 (0)	0/1 (0)
M172	0/1 (0)	0/1 (0)	0/1 (0)	0/1 (0)
Asan 50594	0/1 (0)	1/1 (100)	0/1 (0)	0/1 (0)

*SNP, single-nucleotide polymorphism; MAB, *M. abscessus* subsp. *abscessus*; NT, not tested. CSU, Colorado State University; NIH, National Institutes of Health; MMA, *M. abscessus* subsp. *massiliense*; MBO, *M. abscessus* subsp. *bolletii*; RGM, rapidly growing mycobacteria. †Information collected from available whole genome sequencing data.

Multiple alignment of *rpoB* sequences among available *M. abscessus* subsp. *massiliense* genomes showed the absence of the 2-SNP *rpoB* signature in most strains. However, both SNPs were present in 1 strain not related to an outbreak (1S-151–0930) (Table 2).

Multiple alignment of *secA1* sequences among available *M. abscessus* subsp. *massiliense* genomes showed a G→T substitution at position 820 (by using the *secA1* gene MAB_3580c from the *M. abscessus* subsp. *abscessus* type strain) shared by the Papworth and Seattle cystic fibrosis outbreak strains but not by the soft tissue outbreak strains from Brazil or additional unrelated strains. Further analysis of *secA1* sequences from 12 *M. abscessus* subsp. *massiliense* identified by multitarget sequencing and PCR-based typing (*29**,**38*) showed a G→T substitution at position 820 in 2 strains unrelated to the outbreak (Table 2). Those 2 strains were included among the 4 strains that had the 2-SNP *rpoB* signature. Although the SNPs described for *rpoB* and *secA1* were not 100% specific markers for the outbreak strains, these SNPs could be used for first-level identification of newly isolated strains as possibly being related to cystic fibrosis clusters or soft tissue outbreak strains from Brazil to be confirmed by a second assay.

**Figure 3:**
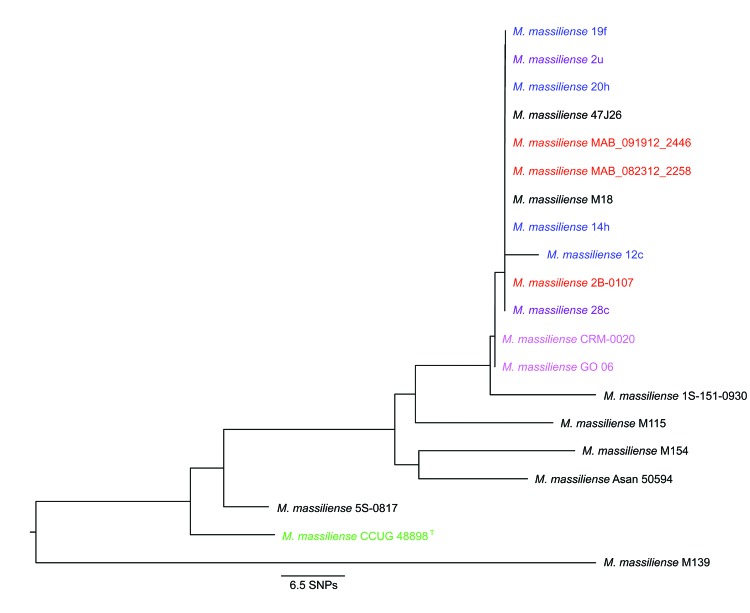
Neighbor-joining phylogenetic tree based on 13-target multilocus sequences types from 20 *Mycobacterium abscessus* subsp. *massiliense* genomes. Electronic PCR was performed on the *M. abscessus* subsp. *massiliense* genomes listed in Table 1 by using primer pairs for 13 housekeeping genes (*cya*, *gdhA*, *argH*, *glpK*, *gnd*, *murC*, *pgm*, *pknA*, *pta*, *pur*, *rpoB*, *hsp65*, and *secA1*), including new primers designed as part of this study. Nucleotide sequences from each gene were concatenated for each genome and aligned by using ClustalW (*31*), and the core alignment was used for construction of a midpoint-rooted neighbor-joining phylogenetic tree by using MEGA (*24*). Strains from an outbreak of *M. abscessus* subsp. *massiliense* infections at a cystic fibrosis center in Seattle, Washington, USA, are indicated in red; strains from an outbreak of *M. abscessus* subsp. *massiliense* infections at a cystic fibrosis center in Papworth, UK, are indicated in blue (cluster 1) and purple (cluster 2); strains from Brazil are indicated in magenta; and the *M. abscessus* subsp. *massiliense* type strain is indicated in green. The longer branch length for Papworth isolate 12c was caused by low-quality nucleotides (single-nucleotide polymorphisms [SNPs]) located at the edge of Velvet contigs.

We also developed a simple MLST protocol that could be used as a second confirmatory assay. Alleles for each of 13 housekeeping genes (*cya*, *gdhA*, *argH*, *glpK*, *gnd*, *murC*, *pgm*, *pknA*, *pta*, *pur*, *rpoB*, *hsp65*, and *secA1*) were extracted and concatenated for each *M. abscessus* subsp. *massiliense* genome (Table 1), and the panel of concatenated sequences was used for construction of a neighbor-joining phylogenetic tree by using MEGA software. The Seattle and Papworth cystic fibrosis outbreak strains grouped together in the tree with cystic fibrosis strain 47J26 and isolate M18 from Malaysia (Figure 3). Thus, partial sequencing of *rpoB* and *secA1* gens, followed by 13-target MLST analysis, could be used to rule out isolates as belonging to these 2 cystic fibrosis clusters.

## Discussion

The implications of this study are extensive. Currently, most experts recommend identifying isolates of *M. abscessus* to subspecies level (*39*). This report further corroborates these recommendations and places even greater pressure on clinical laboratories to fully identify *M. abscessus* subspecies *massiliense*.

Strains from the 2 cystic fibrosis outbreaks showed high-level relatedness (4,264,844 nt core genome alignment size, 11 shared unique SNPs) with each other and major-level relatedness (4,231,390 nt core genome alignment size) with soft tissue epidemic strains from Brazil. Genomic features shared between strains from all 3 outbreaks might make them more transmissible, whether from patient to patient (directly or indirectly as in cystic fibrosis outbreaks) or from a common source, as in soft tissue infections. However, the soft tissue strain from Brazil had the largest number of unique SNPs (86) not shared with either of the cystic fibrosis outbreak strains, harbored an IncP-1β plasmid, and did not show mutational resistance to amikacin or clarithromycin. We speculate that some of these specific genomic traits may be favorable for the successful establishment of epidemic soft tissue infections.

A previous study did not detect a common source or person-to-person transmission of the *M. abscessus* group among cystic fibrosis patients and suggested that it may not be necessary to segregate persons infected or colonized with *M. abscessus* from those who are not infected or colonized (*40*). Our findings emphasize the necessity of screening all isolates of *M. abscessus* subsp. *massiliense* recovered from patients with cystic fibrosis for relatedness to outbreak strains in an effort to prevent future outbreaks. Because of evidence supporting patient-to-patient transmission of multiple different respiratory tract organisms, the Infection Control Guidelines (currently in draft form for public comment) of the United States Cystic Fibrosis Foundation (CFF) (www.cff.org/LivingWithCF/Webcasts/ArchivedWebcasts/Germs/#Infection_Prevention_and_Control_Policy_Update) have been recently changed. Patients with cystic fibrosis are advised not to attend indoor meetings with other cystic fibrosis patients (CFF and Infection Prevention and Control Guidelines 2013). In addition, screening of all cystic fibrosis patients in the United States at least annually for mycobacteria is now recommended (CFF and Infection Prevention and Control Guidelines 2013) to enable early treatment if the organism is detected.

It remains unclear why intercontinental organisms are so closely related. One hypothesis is that direct patient contact led to transmission. The Seattle index case-patient traveled to British Columbia, Canada, before and after acquiring mycobacterial infection, to Oregon before mycobacterial infection, and to Atlanta, Georgia, and Bethesda, Maryland, after mycobacterial infection. However, the patient did not report any contact with other cystic fibrosis patients at these destinations. A second hypothesis is that the mycobacterial strain could have been carried by persons with cystic fibrosis who were clinically well. A third hypothesis is that there was an independent selection of *M. abscessus* subsp. *massiliense* clones in the cystic fibrosis airway milieu on both sides of the Atlantic Ocean toward potentially more transmissible lineages. Availability of additional whole-genome sequencing data tracking the global epidemiology of the *M. abscessus* group may help differentiate between these scenarios. In addition, this data will help delineate global clusters of *M. abscessus* subsp. *massiliense* strains with potentially higher transmissibility.

### Addendum

Recent whole-genome data show deep genetic separation of 3 subspecies, ruling against grouping *M. massiliense* and *M. bolletii* under *M. abscessus* subsp. *bolletii*.

Technical Appendix Single nucleotide polymorphisms shared and unique for subsets of *Mycobacterium abscessus* isolates from cystic fibrosis and soft tissue outbreak localities.
